# Evolutionary Insights and Flowering Regulation of *SPLs* in Coconut Palm

**DOI:** 10.3390/plants14162532

**Published:** 2025-08-14

**Authors:** Runan Chen, Yalan Feng, Jin Zhou, Ying Wang, Fengyi Zhang, Shazia Rehman, Zhuang Yang, Zifen Lao, Hang Xu, Yong Xiao, Jie Luo, Wei Xia

**Affiliations:** State Key Laboratory of Topical Crop Breeding, School of Breeding and Multiplication (Sanya Institute of Breeding and Multiplication)/School of Tropical Agriculture and Forestry, Hainan University, Sanya 572025, China; 21110901000003@hainanu.edu.cn (R.C.); 24110901000039@hainanu.edu.cn (Y.F.); 24220951310206@hainanu.edu.cn (J.Z.); 990811@hainan.edu.com (Y.W.); 20233004553@hainanu.edu.cn (F.Z.); 2020500901000013@hainanu.edu.cn (S.R.); yangzhuang@hainanu.edu.cn (Z.Y.); 22210901000007@hainanu.edu.cn (Z.L.); hang.xu@hainanu.edu.cn (H.X.); jie.luo@hainanu.edu.cn (J.L.)

**Keywords:** *CnSPL*, miR156-targeted, divergence, evolution, flowering

## Abstract

*Squamosa Promoter-Binding Protein Like* (*SPL*) is a critical transcription factor that plays a significant role in regulating plant growth and development. Mining the coconut SPL family offers valuable insights into the regulation of important agronomic traits, including the length of the juvenile phase. In this study, 25 *CnSPLs* were identified and were classified into eight subfamilies. Analysis of gene structure and conserved protein motifs indicated a high conservation of *CnSPLs* within the same subfamilies; however, variations in protein structure and gene length were observed across different subfamilies. Gene expansion analysis indicated that most gene members within subfamilies originated from duplications of the same genomic segment, and transposable element insertion contributed to the divergence of gene sequences within these subfamilies. Characterization of the miR156 target sequence in SPL transcripts revealed that subfamilies IV to VIII contained these sequences, while subfamilies I to III did not. In both coconut and 14 other plant species, some *SPLs* lost their miR156-binding loci due to gene structure variations. The gene expression profiles revealed significant divergence between miR156-targeted and non-targeted *CnSPLs*; the former exhibited low expression levels in the endosperm, while the latter showed comparable expression across all tissues. Notably, *CnSPL15A* demonstrated steadily increasing expression levels in leaves throughout successive leaf primordia and significantly promoted flowering when overexpressed in Arabidopsis. Transient expression assays and 5′ RACE confirmed that *CnSPLs* are targeted by miR156. This study establishes a foundation for investigating the evolutionary characteristics of *CnSPLs* and provides a theoretical framework for analyzing the functions of key *CnSPLs* involved in the coconut flowering control pathway.

## 1. Introduction

*Squamosa Promoter-Binding Protein Like* (*SPL*) is a crucial transcription factor that plays a key role in regulating plant growth and development. The *SPL* genes were first identified in a cDNA library of *Antirrhinum majus* inflorescence, where they were named *SBP1* and *SBP2* due to their ability to bind to the *SQUAMOSA* promoter [1]. SPLs are characterized by the presence of a highly conserved *SQUAMOSA* promoter-binding protein (SBP) domain, which includes two zinc-binding sites and a nuclear localization signal (NLS) [2]. The functions of *SPLs* are remarkably diverse, encompassing nearly every aspect of plant growth and development, and they are involved in processes such as leaf development [3], root development [4,5], shoot architecture [6], phase transition [7,8], flower and fruit development [9,10], as well as gibberellin signaling [11]. Despite the critical roles of *SPLs* in various biological processes, research on this gene family in coconuts is currently limited. Therefore, a thorough exploration and characterization of *SPLs* in this knowledge-limited crop species could provide essential insights into their roles in regulating growth, development, and important agronomic traits.

Numerous studies have identified the functional roles of various *SPLs* as integral components of the *microRNA156*-*SPL* (*miR156*-*SPL*) regulatory modules. This module acts as a critical signaling hub, integrating both endogenous signals and environmental cues to coordinate developmental responses in plants. Within the *miR156*-*SPL* regulatory system, miR156 primarily binds to complementary sequences located in the coding regions or 3′ untranslated regions (3′ UTRs) of *SPLs* [7]. This interaction leads to the post-transcriptional silencing of *SPLs* through mRNA cleavage and degradation or translational repression [3,12]. One of the most significant biological processes regulated by the *miR156*-*SPL* module is flowering, where it facilitates the transition from the vegetative phase by interpreting developmental cues associated with cell division [13,14]. The *miR156-SPL9-DFR* pathway regulates the balance between plant development and stress tolerance by integrating environmental signals, delaying flowering under stress to enhance survival [8]. Aerial bud initiation in switchgrass was regulated by an *miR156-SPL4* module, where *SPL4* acts as a suppressor of both aerial and basal bud formation [6]. In blueberry (*Vaccinium corymbosum*), the *VcMIR156a*-*VcSPL12*-*VcMYBPA1* pathway serves as a key regulator of fruit coloration by controlling anthocyanin biosynthesis and chlorophyll degradation during fruit development [15]. Moreover, in root development, miR156 and its target *SPL10* regulate root meristem activity and de novo shoot regeneration by integrating age cues with cytokinin responses [5]. Analyzing and characterizing the *miR156-SPL* modules could facilitate the identification of key functional regulatory modules involved in plant growth and development.

With the increased availability of high-quality whole genome sequencing, conducting a comprehensive analysis of *SPL* gene families has become more convenient, enabling the exploration of evolutionary characteristics and the identification of key candidate genes involved in plant biological processes [16,17,18]. Characterizing *MtSPL* genes in *Medicago truncatula* has revealed that the *MtmiR156*-*MtSPL* module plays a crucial role in the development of seed pods, particularly in spine formation for seed dispersal [19]. In pepper (*Capsicum annuum* L.), an investigation of the *miR156*-*SPLs* module had revealed its correlation with age-related agronomic traits such as leaf shape and vein number, thus providing insights into the regulation of vegetative phase change [20]. Similar studies have been conducted in *Hordeum vulgare*, *Zanthoxylum armatum*, and various orchid species to identify candidate SPLs involved in regulating abiotic stress responses, flowering, and flower development [17,21,22]. Making evolutionary comparisons between *SPLs* across different genera and families, along with screening and identifying *miR156*-*SPL* modules, will provide a foundation for gaining deeper insights into their functions.

The coconut palm (*Cocos nucifera*) is a quintessential tropical tree, often referred to as the “tree of life” by the people living in tropical regions. It provides a wide array of resources, including food, oil, and materials for construction. The length of the juvenile phase in coconuts is one of the most important agronomic traits, and shortening the time until coconuts reach productive maturity is crucial for early fruit production. Previous research has drawn significant attention to the genetic basis of flowering time control in the coconut palm; however, the key *miR156*-*SPL* module in the aging pathway has yet to be revealed [23]. With the advancement of next-generation sequencing techniques, high-quality genome sequences of the coconut palm have been obtained [24,25]. These resources provide a solid foundation for the genetic exploration of the *miR156*-*SPL* module related to the regulation of the juvenile phase. The primary objective of our research is to systematically analyze the *CnSPL* genes in coconut palms. Specifically, we aim to elucidate the regulatory mechanisms by which miR156 targets *CnSPLs*. In this study, we performed a systematic identification and characterization of *CnSPL* genes, explored and validated the *miR156*-*CnSPL* module, and assessed the gene function of *CnSPL15A*, which is related to the age pathway. This work establishes a basis for investigating the evolutionary characteristics of *CnSPLs* and lays a theoretical foundation for analyzing the functions of key *CnSPLs* involved in the coconut flower control pathway.

## 2. Materials and Methods

### 2.1. Data Sources and Sequence Retrieval

The coconut genome sequence, gene protein sequences, and the transcriptome datasets used in this study were generated from our previous research [24,26]. The RNA-seq SRAs (CRA004778, https://ngdc.cncb.ac.cn/gsa (accessed on 10 October 2023)), created in our previous research, were used in this study. The transcriptome dataset includes RNA-seq data for five types of tissues—leaf, flower, stem, endosperm, and mesocarp. Additionally, the genome sequence, gene model information, transcript, and protein sequences of the other 14 species, *Amborella trichopoda*, *Daucus carota*, *Solanum tuberosum*, *Vitis vinifera*, *Malus domestica*, *Citrus sinensis*, *Arabidopsis thaliana*, *Dioscorea alata*, *Phoenix dactylifera*, *Elaeis guineensis*, *Musa acuminata*, *Ananas comosus*, *Brachypodium distachyon*, and *Oryza sativa,* were retrieved from the Phytozome website (http://www.phytozome.net/ (accessed on 20 September 2024)) and the National Center for Biotechnology Information (NCBI) website (https://www.ncbi.nlm.nih.gov/ (accessed on 20 September 2024)).

### 2.2. Genome-Wide Identification of SPLs Genes

The hidden Markov model (HMMER) profile of the SBP domain (Pfam accession: PF03110) was obtained from the Pfam database (http://pfam.xfam.org/(accessed on 20 September 2023)) and used as a query to search for SPL members in coconut and 14 other species mentioned above. The peptide sequences of the SBP domain were extracted based on the results of the HMMsearch analysis. Multiple sequence alignments on the SBP sequences were conducted using ClustalW with default parameters. The phylogenetic tree of *CnSPLs* and *AtSPLs* was constructed using the Maximum Likelihood method based on the JTT matrix-based model in MEGA 7.0 [27], and visualized in the online software iTOL (v6) [28]. *SPL* genes from coconut and 13 other species (excluding Arabidopsis) were then subjected to BLAST (v2.14.1) searches against *AtSPLs* to identify the best homologous hits. The BLAST results were also utilized to assign *SPL* subfamilies. The gene list and subfamily information for the 15 species were included in Table S1.

### 2.3. Evolutionary Analysis of CnSPL Genes

Multiple sequence alignments for *CnSPLs* were conducted to construct a phylogenetic tree using MEGA 7.0 with 1000 bootstrap replicates [27]. Additionally, a phylogenetic tree of coconut palm and fourteen other species, developed in our previous research, was incorporated in this study [29]. The phylogeny was inferred using RAxML v8 with the PROTGAMMAJTT model, employing 1000 bootstrap replicates and 140 single-copy genes [30].

All protein-coding genes from coconut were aligned using BLAST against the coconut protein-coding gene database, with a cutoff of 1 × 10^−5^. The BLAST results were processed using the software MCScanX (v1.1) to identify homologous chromosomal regions within coconut palm and between species that contain *SPL* genes [31]. Duplicated gene pairs of *CnSPLs*, was well as homologous gene pairs of *CnSPLs* and *AtSPLs* within homologous genomic segments, were identified based on the following three criteria: (a) the alignment covered >80% of the longer gene; (b) the aligned region had an identity >80%; and (c) only one duplication event was counted for the tightly linked genes. The duplicated gene pairs and homologous genomic segments were visualized using TBtools (v1.106) software [32].

### 2.4. Promoter Analysis and Gene Expression Pattern Analysis Based on Transcriptome Datasets

The 2000 bp upstream sequences beginning from the start codon were extracted from CnSPLs. Promoter motif analysis was performed using the online tools PlantCare (2023 release, http://bioinformatics.psb.ugent.be/webtools/plantcare/html/) and TSSP (26 October 2016, http://www.softberry.com/) [33]. Potential TATA-boxes, CAAT-boxes, and motifs related to tissue-specific expression and phytohormone responsiveness were identified with TBtools.

The RNA-seq SRAs (CRA004778, https://ngdc.cncb.ac.cn/gsa) generated in our previous study were utilized in this research. This transcriptome dataset encompasses RNA-seq data from five tissue types: leaf, flower, stem, endosperm, and mesocarp. FPKM values were calculated as outlined in our earlier work, using Hisat2 (v2.2.0) for read mapping and Stringtie (v2.2.1) for isoform assembly [29,34].

### 2.5. Identification the Complementary Loci of miR156 in SPL Transcripts

Transcripts for SPL genes were identified in both coconut palms and the other 14 species based on gene model information and available transcriptome datasets. The mature miR156 sequence was obtained from our previous research [35]. The computational software psRNAtarget (2017 release) [36] and TargetFinder (v1.6) [37] were used to predict the targeted loci of miR156.

### 2.6. Analysis of the Expression Patterns of CnSPLs Using Reverse Transcription Quantitative Polymerase Chain Reaction (RT-qPCR) Assays

Three five-year-old coconut palm trees displaying their first spathes were selected to analyze the expression levels of *CnSPL* genes. The visible leaves from successive primordia were assumed to be in the various stages of development, transitioning from the juvenile to the adult stage. Leaves were collected from the bottom to the top, named as L1 (the first leaf) to L10 (the tenth leaf). The leaves in the same order from three selected coconut trees were designated as biological replicates. The total RNA was extracted according to our previous protocol [38]. For each sample, first strand cDNA was synthesized in accordance with the manufacturer’s instructions (HiScript III 1st Strand cDNA Synthesis Kit, R312, Vazyme, Nanjing, China). Real-time qPCR was performed following the ChamQ Universal SYBR qPCR Master Mix kit protocol (Vazyme, China). All PCR reactions were performed using an ABI 7900HT machine with the following program: 95 °C for 30 s, then 40 cycles of 95 °C for 5 s, 55 °C for 15 s, and 68 °C for 20 s, in 384-well clear optical reaction plates (Applied Biosystems, Foster City, CA, USA). The primers used for RT-qPCR are listed in Table S2a.

### 2.7. Transient Expression of CnSPL15A in Tobacco Epidermal Cells for Subcellular Localization

The full-length coding sequences of *CnSPL15A* were amplified using primers listed in Table S2a. We used the Uniclone One Step Seamless Cloning Kit (Genes and Biotech Company, Bejing, China) to construct the OE-expression *CnSPL* vector—pc1300-35S-CnSPL15A-eGFP. The pc1300-35S-eGFP plasmid was linearized by digesting with *Sal* I and *Kpn* I. Homologous recombination was used to link the amplified *CnSPL15A* to the linear pc1300-35S-eGFP plasmid. Recombinant plasmid-positive clones were screened and amplified in *Escherichia coli* DH5α. The bacteria were transformed via heat shock and cultured overnight at 37 °C in LB broth supplemented with kanamycin (50 µg/mL).

The recombinant plasmids were transformed into *Agrobacterium tumefaciens* strain GV3101 by heat shock. For the Agrobacterium cultures, we used LB medium and incubated them at 28 °C. The Agrobacterium cultures containing the 35S::eGFP, 35S::CnSPL15A:eGFP, and 35S::OsGhd7:RFP fusion constructs (the latter serving as a positive control for nuclear localization) were pelleted and resuspended in the infiltration medium. The infiltration medium consisted of the following components: Murashige and Skoog salts (4.3 g/L), sucrose (30 g/L), 2-(N-Morpholino) ethanesulfonic acid (50 mM, pH 5.6), and acetosyringone (100 μM). The bacterial suspension was adjusted to an optical density (OD_600_) of 1.0 before being infiltrated into the abaxial surface of fully expanded tobacco (*Nicotiana benthamiana*) leaves from 4-week-old plants. After infiltration, the plants were kept in the dark at 26 °C to promote optimal conditions for transformation and expression of the introduced constructs. GFP signals were detected at time intervals of 48–72 h post-infiltration using a confocal microscope (LSM980, Zeiss, Jena, Germany).

### 2.8. Transient Expression of miR156 and SPL-eGFP Fusion in Tobacco Epidermal Cells for Target Sequence Validation

The primary sequence of *miR156* was used based on our previous research [35], which contains the full-length hairpin structure and was supported by the transcriptome dataset used in this study. We amplified pri-miR156 and cloned it into the pc1300-35S-flag vector. Both of the PCR product and the plasmid were digested with *Hind* III and *Sal* I (Nova, Hehui, Haikou, China). Two representative miR156-targeted sequences in *CnSPLs*, referred to as Seq1 and Seq2, were linked to pc1300-35S-eGFP plasmid, which was digested with *Kpn* I (Nova, Hehui, Haikou, China). This resulted in the formation of a fused protein that was expressed in the same open reading frame as the eGFP protein. The primers used for the primary miRNA and the targeted sequences in *CnSPLs* are listed in Table S2b. The transient expression assay was conducted as described above. GFP signals were detected at time intervals of 48 to 72 h post-infiltration using Handheld UV Lamp (3260RB, LUYOR, CA, USA). The digital values of GFP signals were obtained from the inoculated spots using a confocal microscope (SESIS, LMS980), and the values were collected for three biological replicates in each transient expression combination.

### 2.9. RLM-5′ RACE for miR156-Targeted SPL Validation

Total RNA extracted as described in the above was used for RLM-5′ RACE. The RNA was ligated to an RNA adapter by T4 RNA ligase (NEB M0437, Ipswich, MA, USA) in a reaction mixture containing 0.5 U/μL of T4 RNA Ligase, 4 U/μL RNAse inhibitor, and 1 mM ATP. Subsequent steps were performed according to the manufacturer’s guide for the GeneRacer kit (Invitrogen, Carlsbad, CA, USA). The first PCR was conducted using an outer CnSPL15A-specific primer: 5′-ACTACTGCCAGCCCCAGTGAC-3′. The second PCR reaction utilized the product from the first PCR along with an internal CnSPL15A-specific primer (5′-GCCTATGTCATGCTGGATTTCAT-3′). After amplification, the 5′-RACE products were gel-purified and cloned, and at least eight independent clones were randomly chosen and sequenced.

### 2.10. Plant Transformation and Transgenic Plants Phenotype Investigation

The OE-expression *CnSPL15A* vector, designated as pc1300-35S-CnSPL15A-eGFP, was utilized for the transformation of Arabidopsis. The construct was introduced into the *Agrobacterium tumefaciens* strain GV3101 using the freeze–thaw method. Transgenic plants were generated via the floral dipping method [39] and screened on half-strength Murashige and Skoog plates supplemented with 50 mg/mL of hygromycin. Flowering time was assessed by counting the total number of leaves (including both rosette and cauline leaves) and recording the number of days until flowering commenced (indicated by the appearance of flower buds). Ten transgenic plants from independent lines were grown under a 16 h light/8 h dark cycle at 22 °C for phenotypic investigation.

For the T_2_ generation transgenic plants, samples were taken for total RNA extraction following the aforementioned protocol. Three independent lines exhibiting a distinct early flowering phenotype were selected. For each sample, first-strand cDNA synthesis and Real-time qPCR were conducted according to the protocols described earlier. The primers used for RT-qPCR are listed in Table S2a.

### 2.11. Statistical Analysis

For the statistical analysis of the data obtained from the above RT-qPCR assays, phenotypic comparisons between transgenic and wild-type Arabidopsis, and digital eGFP fluorescence values acquired from confocal microscopy, we employed Student’s *t*-test. This test was used to assess the significance of differences between the means of the two groups (transgenic and wild-type). Prior to analysis, data were checked for normality and homogeneity of variances to ensure the appropriateness of the *t*-test. A *p*-value of less than 0.05 was considered statistically significant. All statistical analyses were performed using the SPSS software (v25), and results were presented as mean ± standard error of the mean (SEM).

## 3. Results

### 3.1. Gene Characters of the SPL Gene Family (CnSPLs) in Coconut Palm

**Figure 1:**
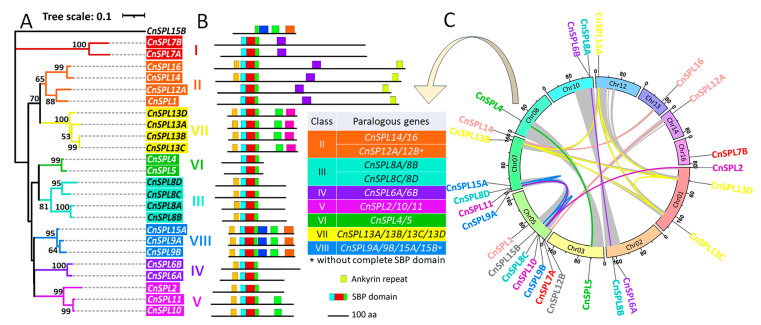
Evolutionary characters of *CnSPLs* in coconut palm genome. (**A**) The phylogenetic tree of *CnSPLs* was constructed using the Maximum Likelihood method based on the JTT matrix-based model, along with a multiple sequences alignment of SBP domain sequences in MEGA 7.0. (**B**) The conserved motif of CnSPL proteins were visualized based on the analysis results from the online MEME software. (**C**) The genomic locations of *CnSPLs* were identified within the duplicated genomic segments. Duplicated genomic segments and duplicated *CnSPLs* were determined through MCScanX analysis and visualized by TBtools. The asterisk indicates that CnSPL15B and CnSPL12B lack a complete SBP domain or a detectable SBP domain, although they are homologous to the other CnSPLs in the paralogous genomic segments. CnSPL12B (AZ05G0097630) displayed high homology with CnSPL12A (e-value < 1 × 10^−5^).

To identify the *SPL* genes in coconut, we conducted hidden Markov model (HMMER) search against the coconut genome protein database using the SBP domain (PF03110) as a seed. Conserved SBP domains were identified by analyzing and verifying the corresponding sequences against known SBP domain sequences in the database. Twenty-five CnSPL proteins with SBP domains were identified and designated based on their best matches to AtSPL proteins (Table S1). Because of the high divergence of CnSPL proteins, the conserved SBP domain sequences were obtained to construct the phylogenetic tree. Among these *CnSPL* members, *CnSPL11* had an incomplete SBP domain sequence and was used as an outgroup member (Figure 1A). Phylogenetic analysis revealed that the remaining 24 CnSPL proteins containing complete SBP domains were classified into eight distinct subfamilies, based on the established subfamily categorization of their closest homologs—the AtSPL proteins identified through best-hit sequence alignment. Further construction of phylogenetic tree including *CnSPLs* and *AtSPLs* also supported the subfamily classification (Figure S1A). Among these subfamilies, I, III, IV, and VII contain more *CnSPLs* than their *AtSPL* counterparts, while subfamilies II and V had same gene numbers. Conversely, subfamily VI has fewer *CnSPLs* than found in Arabidopsis. Topological analysis revealed closer phylogenetic relationships among specific subfamilies, with three distinct clusters emerging, subfamilies II/VII, III/VI, and IV/V/VIII, forming cohesive evolutionary groups (Figure 1A).

To shed light on evolutionary characters of the CnSPLs protein sequences, conserved motifs were detected by the MEME online software (v4.12). Among all the conserved motifs identified, the majority of *CnSPLs* within the same families exhibited similar distributions of motif types. Additionally, more than one motif was shared among different, aside from the SBP domain (Figure 1B). All CnSPL proteins, with the exception of CnSPL15B, contained the SBP domains. However, the positioning of the SBP domain varied among different CnSPL proteins. Notably, *CnSPLs* in subfamily VII and VIII had their SBP domain located closer to the N-terminus compared to other members. Most *CnSPLs* contain an additional conserved motif preceding the SBP domain, with the exceptions being members in subfamily I and VI, as well as *CnSPL18*/*24* in subfamily II. Although CnSPL15B had an incomplete SBP domain sequence, it shares the same four motifs identified in subfamily VIII. Additionally, a specific Ankyrin repeat motif was detected in subfamily II, while the remaining motifs were shared among multiple subfamilies, albeit with divergent combinations.

The expansion of *CnSPLs* widely occurred through segmental duplication, which were detected in seven subfamilies except for subfamily I (Figure 1C). The expansion of CnSPL gene members in these subfamilies resulted from segmental duplication, triplication, or tetraplication. Duplicated genomic segments with *CnSPLs* were the dominant type and detected in subfamilies II, III, IV, and VI, with a total of six pairs of duplicated gene pairs. In subfamily II, *CnSPL12A/12B* were located within paralogous segments, while *CnSPL12B* was not included in the phylogenetic analysis because its SBP domain was lost due to transposable element (TE) insertion. Additionally, triplication and tetraplication events were detected in subfamily V and subfamilies VII/VIII, respectively. CnSPL15B protein had similar conserved peptide motifs with *CnSPL* members in subfamily VIII, and the genomic duplication result also confirmed that these genes were derived from same ancestor segment.

The identification of homologous genomic segments between coconut palm and Arabidopsis also showed that *CnSPLs* and *AtSPLs* in subfamilies I, II, VII, and VIII exhibit collinearity segments (Figure S1A). In subfamily I, *CnSPL7A* and *CnSPL7B* exhibited no evidence of originating from genomic duplication, with only *CnSPL7B* found in homologous segments alongside *AtSPL7*. In contrast, *CnSPL14* and *CnSPL16* in subfamily II were located in homologous segments corresponding to *AtSPL14* and *AtSPL16*. The genomic segments containing *CnSPL13B* and *CnSPL13D*, which belong to subfamily VII, showed a collinear relationship with the segments of *AtSPL13A*. Furthermore, the four paralogous genes—*CnSPL9A*, *CnSPL9B*, *CnSPL15A*, and *CnSPL15B*—exhibited a collinear relationship with the genomic segments associated with *AtSPL9* and *AtSPL15* (Figure S1B). These homologous segments share a common ancestral origin, suggesting that the homologous *SPLs* may have similar gene functions.

### 3.2. Evolutionary Character of miR156-Targeted Loci in SPLs

**Figure 2:**
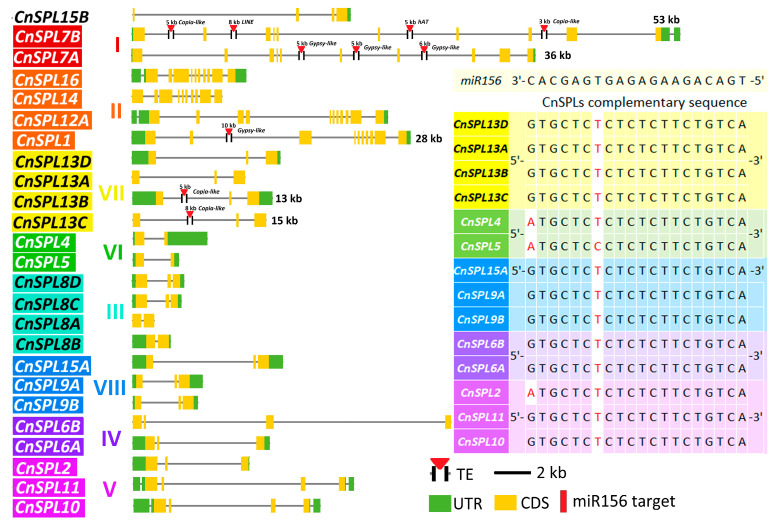
Gene structure and complementary sequence loci of miR156. Gene structures of *CnSPLs* displayed according to gene model information and ISOseq data modification. The transposable element located in the *CnSPL* introns was analyzed by RepeatMasker. The complementary sequence of miR156 in *CnSPLs* was analyzed by psRNATarget and BLAST to the coconut genome sequence to determine its location. The mature miR156 sequence was identified based on the conserved miR156 found in the miRbase database and our previous research.

To further characterize the evolutionary features of *CnSPLs*, the gene structure feature and the complementary loci of miR156 were analyzed. In accordance with the phylogenetic results and gene expansion results, the number and distribution of exons were conserved within subfamilies (Figure 2). Moreover, the number of exons varied among different subfamilies, ranging from 2~3 in most subfamilies to 10~12 in subfamily I and II, with exceptions for *CnSPL11*/*20* (4 exons) and *CnSPL9*/*14* (6 exons). Additionally, the intron lengths varied significantly both within the same family and between different families. *CnSPL7* and *CnSPL25* in subfamily I exhibited unusually long gene lengths of 53 kb and 36 kb, respectively, due to transposable element (TE) insertions within introns. *CnSPL3* (15 kb), *CnSPL12* (13 kb), and *CnSPL17* (28 kb) also have one TE insertion each, resulting in significantly longer lengths compared to other members in the same subfamily.

*SPLs* are well-known targets of *miR156*; however, not all *SPL* genes are regulated by this microRNA. To identify and analyze the presence of miR156 complementary binding sites, which are involved in forming the *miR156*-*SPL* regulatory module, we conducted an analysis of the SPL target sites that interact with miR156. Among *CnSPLs*, members of subfamilies I–III lack miR156 target sites, whereas those in subfamilies IV to VIII possess conserved miR156 complementary loci (Figure 2). The locations of the targeted sequences were in the exon regions for the *CnSPLs* in subfamilies VII and VIII, mostly located in the last exon. For subfamilies V and VI, the complementary loci located in the 3′UTR regions. *CnSPL1* in subfamily I has lost its miR156-targeted locus in its transcript sequence, although it still exists in the downstream region of the gene. Further analysis of these targeted sequences indicated that most of them exhibit strong complementarity with the miR156 mature sequence, containing only one mismatch, with the exception of *CnSPL1*, *CnSPL6*, and *CnSPL19* with two mismatches.

**Figure 3:**
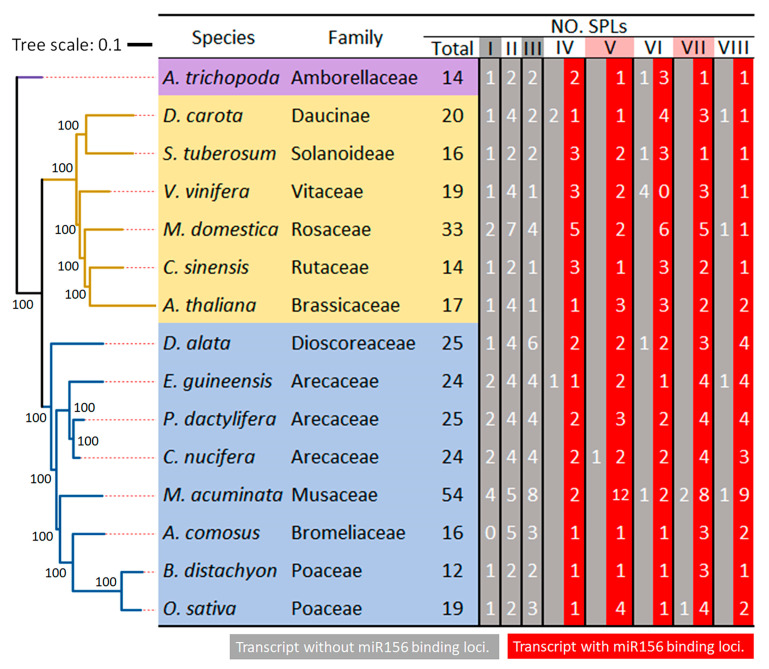
The phylogenetic tree, gene numbers, and miR156-binding loci of *SPLs* in coconut and fourteen other species within the context of angiosperms. The ML phylogenetic tree of these 15 species was constructed using 140 single-copy genes detected by the OrthoFinder (v3.1.0) [41], a tool that was also utilized in our previous research [35]. The *SPL* gene numbers and miR156-binding loci were determined by the method used for coconut in this study, specifically employing gene-derived transcript sequences as input data for miR156 target prediction through two computational tools: psRNAtarget [36] and TargetFinder [37].

To further characterize the evolutionary feature of *SPLs*, we conducted a comparative analysis of the number, classes, and miR156-targeted loci of *SPLs* across coconut palm and other 14 species, including a basal extant flowering plant *Amborella trichopoda*, six dicot species, and eight monocot species (Figure 3). The *SPLs* for the other fourteen plant species were identified using the same method described applied to coconut and are listed in Table S1. The numbers of *SPLs* ranged from 12 in *B. distachyon* to 54 in *M. acuminata*, with seven species having *SPL* numbers fewer than twenty *SPLs* and two species exceeding thirty (Figure 3). *M. acuminata*, which has the highest number, is thought to be the result from the two whole genome duplication events (denoted as α and β) [40]. *A. trichopoda* is one of the species with the fewest *SPL* genes, possessing a total of 14 SPLs distributed among most subfamilies, with one to two members in each, except for subfamily VI. The same number of 14 SPLs was also found in *C. sinensis*, which contains one to three SPLs in each subfamily. Additionally, the number of SPLs in the eight subfamilies varies among species, particularly in subfamily II (ranging from two to seven), III (from one to eight), and V (from one to twelve).

In the analysis of miR156-binding loci, the *SPLs* from the 15 species showed conservation, with no complementary loci detected in subfamilies I/II/III. However, complementary loci were present in almost all members in the remaining subfamilies (Figure 3). Some species did lose a few miR156-targeted loci in specific subfamilies. In subfamily IV, *D. carota* and *E. guineensis* lost miR156-targeted loci in two and one SPLs, respectively. In subfamilies VI and VIII, four species had SPLs that lost their complementary miR156 loci, while one species lost loci in subfamilies V and VII. Additionally, six species maintained all members in subfamilies IV to VIII with miR156-targeted loci, whereas other species exhibited one to four *SPLs* that lacked these loci.

### 3.3. The Divergent Expression Patterns of CnSPLs

**Figure 4:**
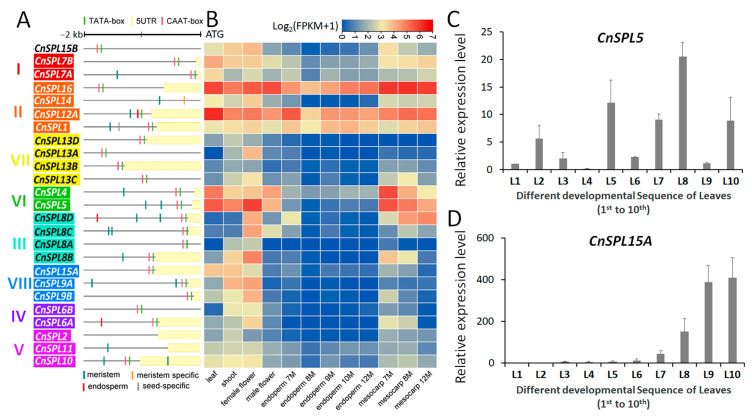
Promoter character and expression patterns of *CnSPLs*. (**A**) Regulatory motifs in *CnSPL* promoters. The 2000 bp upstream sequence from the ATG start codon were marked with the conserved promoter motif and the tissue-specific expression motifs predicted using the Plantcare (2023 release) and TSSP software (2016 release), as well as 5′UTR region derived from transcriptome datasets. (**B**) A heatmap of *CnSPL* expression based on log2-transformed mean FPKM for coconut leaf, shoot, endosperm, and mesocarp tissues was generated using the transcriptomes used in this study. The expression levels of *CnSPL5* (**C**) and *CnSPL15A* (**D**) were detected via an RT-qPCR assay in the different developmental leaves from the bottom to the top of the coconut tree (from successive leaf primordia). For each leaf, three five-year-old trees with their initial flowering were chosen as biological replicates. A total of ten leaves were selected. The previously reported housekeeping gene *CnACT* was used as a reference gene [43].

*CnSPLs* had divergent distribution of regulatory motifs in their promoter region both within and between subfamilies. For the conserved TATA-box and CAAT-box, most *CnSPLs* had these typic motifs with their upstream sequences started from the start codon, except for *CnSPL2*, *CnSPL14*, and *CnSPL16A* (Figure 4A). The motifs related to tissue specific expression, such as meristem, endosperm, and seed expression, were detected in 13 *CnSPLs*. The motif related to meristem expression was detected most frequently, with 12 out of 13 genes containing this motif and covering six subfamilies. The endosperm-specific expression motif was identified in *CnSPL6A*, *CnSPL2A*, and *CnSPL18D*, while the seed-specific motif was only detected in *CnSPL1*.

Further exploration of gene expression profiles indicated that *CnSPLs* in the same subfamily tend to have similar expression patterns, such as *CnSPL23*/*24* in subfamily II (Pearson correlation coefficient (PCC): 0.54, *p* < 0.05) and *CnSPL4*/*5* in subfamily VI (PCC: 0.66, *p* < 0.05) (Figure 4B). Additionally, *CnSPL12/23/24* in subfamily II had high expression across all analyzed tissues, while the remaining genes, such as *CnSPLs* in subfamilies IV, V, VII, and VIII, shared similar expression pattern and had relatively low expression in male flower and endosperm.

The *miR156*-*SPL* module is a well-known component of the age pathway involved in the control of flowering. In *Arabidopsis*, miR156 targets *AtSPL3*, *AtSPL4*, and *AtSPL5* in subfamily VI play important roles in floral transition, while *AtSPL9* and *AtSPL15* in subfamily VIII contribute to the juvenile-to-adult vegetative transition [42]. *CnSPL5* and *CnSPL15A*, which are homologous to *AtSPL5* and *AtSPL9/AtSPL15,* were selected for expression pattern analysis across different developmental stages. Five-year-old coconut palm trees displaying their first spathe were selected to analyzed the expression levels of *CnSPL5* and *CnSPL15A* (Figure 4C,D). The gene expression of *CnSPL5* in successive leaf samples exhibited a pattern of three gradual increases followed by decreases, with no clear correlation to the age of the leaves (from the bottom to the top) (Figure 4C). However, *CnSPL15A* exhibited a distinct patten in its expression level as the leaf aged, with a significant increase observed starting from the seventh leaf, followed by a continuously rise in expression levels from the eighth to the tenth leaves (Figure 4D). *CnSPL15A* is considered as an age-related member of the *CnSPL* family (Figure 4D).

### 3.4. CnSPL15A Is Targeted by miR156 by Transcript Cleavage

**Figure 5:**
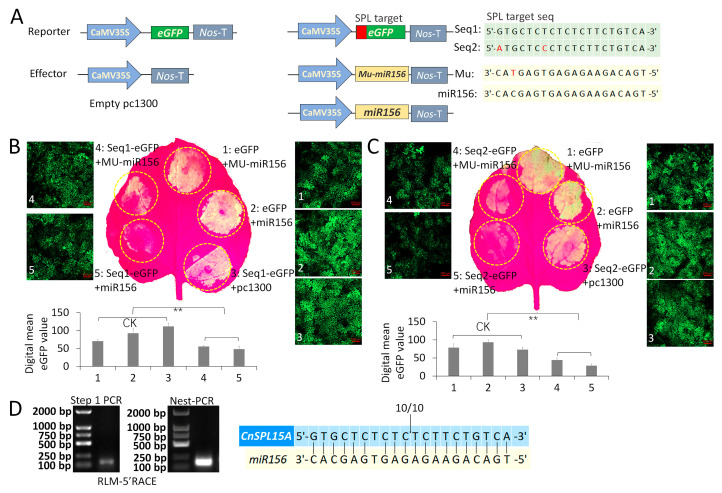
MiR156 targets complementary binding sites within the transcripts of *CnSPL* genes. (**A**) The diagram of vectors used in miR156 targets validation assay. The primary sequences of miR156 and MU-miR156 (which has a one-nucleotide change in the mature miRNA region compared to iR156) were cloned into the pc1300-35S vector to generate the effector constructs. The sequence containing complementary sequences of miR156 mature sequences Seq1 and Seq2 were linked to the eGFP-reporter vector pc1300-eGFP in the same open reading frame of eGFP and with a start codon in the front. (**B**,**C**) GFP signals in transiently transformed tobacco leaves were detected by Handheld UV Lamp (3260RB). Three controls (CK)—35S::eGFP+Mu-miR156, 35S::eGFP+miR156, and 35S::Seq1/2-eGFP+empty clone—were used. The tobacco leaves were sampled for confocal microscope (SESIS, LMS980) detection and digital values of eGFP were obtained with same capture parameters for all samples. The error bars represent the standard error of the mean, reflecting the variability of the digital GFP values collected from three biological replicates for each transient expression combination. ** *p* < 0.01 (Student’s *t*-test). (**D**) The miR156 cleavage sites in *CnSPL15A* mRNA were determined by RLM-5′ RACE. Step 1 PCR used the out primer of the RNA adaptor provided by the GeneRacer kit and the *CnSPL15A* specific out primer to amplify the presupposed cleavage *CnSPL15A* mRNA. The nest PCR used the PCR product purified from Step 1 and inner primers of the RNA adaptor and *CnSPL15A*. The vertical lines represent the nucleotides that base-pair with miR156. The arrows indicate the position of the cleavage site in *CnSPL15A* mRNA.

A transient expression assay was conducted using green fluorescent protein (GFP) as a reporter gene to investigate the regulation of target genes by miR156. This involved selecting conserved complementary sequences (Seq1 and Seq2) that contained one or two nucleotide mismatches to the miR156 sequence, based on the miR156-targeted sequences analysis (Figure 5A). In the transient expression assay, the pri-miR156 overexpression construct was co-expressed with a reporter construct containing the GFP gene linked to the CnSPL target sequences (Seq1 and Seq2). Three controls (CK)—35S::eGFP+Mu-miR156, 35S::eGFP+miR156, and 35S::Seq1/2-eGFP+empty clone—were used. The results showed a significant decrease in GFP signals upon the overexpression of pri-miR156 compared to the control, indicating that miR156 effectively down-regulates the fusion GFP containing a target SPL sequence (Figure 5B,C). Additionally, digital quantification of GFP signal from confocal microscopy further confirmed a significant decrease between the control samples and the experiment sets.

To assess the specificity and efficiency of miR156 binding to its target sequences, a mutated version of the pr-miR156 (MU-miR156) was included, which features a one-nucleotide change in the mature miRNA region. The results indicated that while MU-miR156 resulted in a weaker GFP signal compared to the control, it still exhibited a slightly stronger signal than the normal pri-miR156. This suggests that even with a single nucleotide mutation, MU-miR156 retains some regulatory capability, albeit reduced. Furthermore, both CnSPL target sequences (Seq1 and Seq2) were significantly down-regulated by miR156, further confirming their status as direct targets of this miRNA (Figure 5B,C).

To further validate the target, we mapped the miRNA cleavage sites in one of their common targets (*CnSPL15A*) by RNA ligase-mediated 5′ rapid amplification of cDNA ends (RLM-5′ RACE) (Figure 5C). From the two round PCR amplification, a specific amplicon that covered the cleavage site of CnSPL15A was obtained (Figure 5C left). The sequencing results of randomly chosen clones revealed that the cleavage occurred between the 10th and 11th base pair of the miRNA target site (Figure 5C right). This indicates the CnSPL15A transcripts were down-regulated by miR156 through cleavage at the conserved the 10th/11th base position, as reported in other studies [44,45].

### 3.5. CnSPL15A Localized to the Nucleus and Affected the Vegetative Phase Change

**Figure 6:**
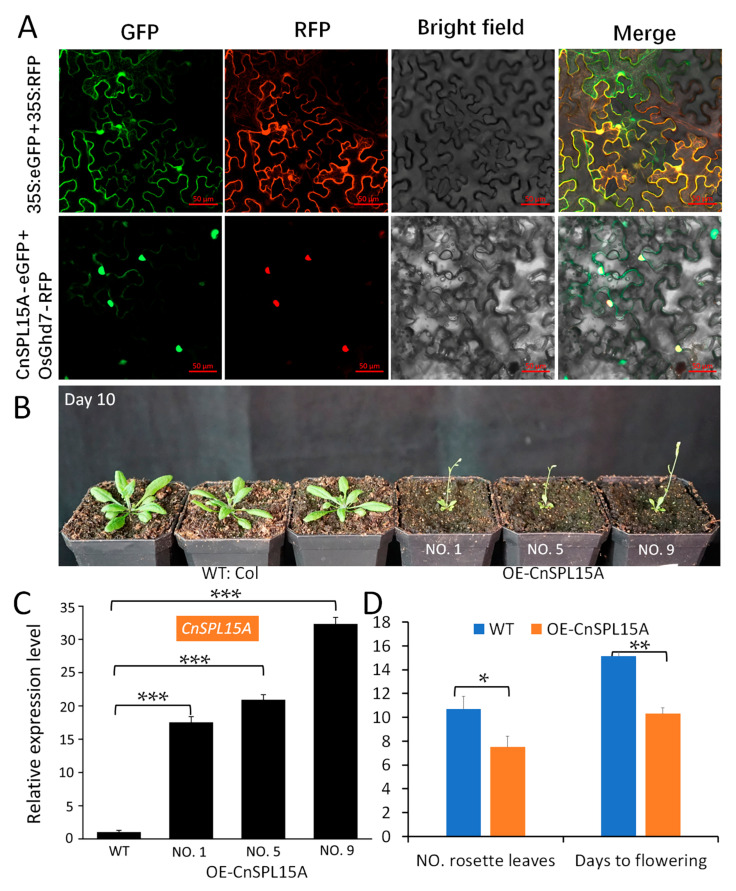
Nucleus-localized CnSPL15A shortens flowering time when overexpressed in Arabidopsis. (**A**) The CnSPL15A protein is localized to the nucleus; (**B**) The wild-type (WT) and OE-CnSPL15A Arabidopsis exhibited distinct variations in flowering time, with ten plants from each line serving as biological replicates for the phenotypic investigation. (**C**) The expression level of *CnSPLs* in WT and OE-CnSPL15A plants were assessed using RT-qPCR assay, with three plants from each line used as biological replicates. (**D**) The phenotype of NO. rosette leaves and days of flowering between WT and OE-CnSPL15A plants. The fusion proteins 35S::CnSPL15A:eGFP and the nucleus marker 35S::OsGh7:RFP were transiently expressed in tobacco epidermal cells. GFP signals were observed using a confocal microscope (SESIS, LMS980) at time intervals of 48 to 72 h after infiltration. GFP: green fluorescence; RFP: red fluorescence; Bright field: visible light; Merge: visible light merged with fluorescence. Scalebars: 50 μm. The reference gene used for RT-qPCR is *AtACT8* (AT1G49240). *** *p* < 0.001, ** *p* < 0.01, * *p* < 0.05 (Student’s *t*-test).

CnSPL proteins were thought to be transcription factors that function primarily in the nucleus. The subcellular localization of *CnSPL15A* was determined using transiently expressed CnSPL15A-GFP fusion proteins in tobacco epidermal cells. The GFP signals were compared with the previously reported nucleus—localized OsGhd7-RFP. Fluorescence signals for CnSPL15A-GFP were specifically observed in the nucleus (Figure 6A).

To validate the function of *CnSPL15A*, OE-CnSPL15A transgenic lines were developed in Arabidopsis (Figure 6B). The T_2_ homologous OE lines exhibited a significant early flowering phenotype, characterized by small, serrated leaves from the first true leaf. The bolting date occurred approximately 10 days after transplanting the Arabidopsis seedlings. The RT-qPCR assay revealed that these lines had significantly higher expression levels of *CnSPL15A* compared to wild-type plants (Figure 6C). Additionally, the average number of rosette leaves in OE-CnSPL15A lines was significantly lower than in wild-type Arabidopsis, averaging 6 to 7 leaves, while wild-type plants typically had more than 10 leaves. Overexpression of *CnSPL15A* also significantly promoted the transition to the reproductive phase; flowering occurred 10 days after planting in soil, which was a significant advancement compared to the wild-type plants (Figure 6D).

## 4. Discussion

*SPL* is a crucial transcription factor that participates in various aspects of plant biological processes, and *miR156*-*SPL* modules are a well-characterized hub in regulatory pathways. In this study, we classified the 25 *CnSPL* genes in coconut into eight subfamilies, with members of the same subfamily primarily arising from segmental duplications on chromosomes. These genes exhibited conserved characteristics in terms of gene structure, composition of protein conserved domains, miR156 target binding sites, and expression patterns. However, some members displayed variations in gene region length and the loss of miR156 target binding sites, which may lead to functional diversification. Based on the gene functions identified in Arabidopsis, we selected and validated *CnSPL15A*, which is involved in regulating flowering time, indicating the conservation of gene function across species. This research provides comprehensive genomic information and important experimental evidence for exploring the *miR156*-*SPL* module in coconuts.

*SPLs* play a critical role in various biological processes, which is attributed to their functional diversification. The *CnSPL* genes in coconuts exhibit significant structural and functional differences across various subfamilies, including genic length, protein domain, and miR156-targeted loci. Even for the SBP domain, evolution and degeneration were detected: CnSPL11 has an incomplete SBP domain, while a homologous gene of CnSPL24, CnSPL26, completely lacks the SBP domain. Other genome-wide characterization of SPL genes indicated the same subfamilies classification, similar gene structure, and conserved protein motifs within the subfamily, suggesting conserved functional divergence for these SPLs [19,21,46,47]. In coconut and these plant species, *SPLs* in same families were composed of same protein motif types, and while subfamily-specific protein motifs were also detected, such as the specific Ankyrin repeat motif that was detected in subfamily II, which makes the proteins in this subfamily much larger than other SPLs [48]. These findings indicate that the SPL gene family are conserved in several aspects between different species, while functionally diversification continues to lead to their involvement in a broad range of biological regulatory processes.

A key feature contributing to functional divergence in *SPLs* is whether they are targeted by miR156. In this study, we characterized *SPLs* in 15 species regarding this trait, which aligns with previous research indicating that 11 *AtSPLs* are targeted by miR156 [49]. Notably, subfamilies I-III lack miR156 target sequences, while subfamilies IV-VIII contain these sequences across all analyzed species. This establishes a foundational basis for the targeted regulation of SPL by miR156. Regarding the conservation of the target sequence, the 21-base pair region shows only minor variations, with one to two base differences in some copies. Previous studies have reported that cleavage usually occurs between the 10th and 11th base of the miRNA target site [44,45]. Despite being targeted by miR156, the regulatory traits associated with miR156-SPL interactions are remarkably diverse and covered many biological processes [50,51]. This diversity is largely attributed to the tissue-specific expression of miR156 and the subsequent targeted SPL transcripts. Moreover, variations in conserved protein motifs among different *SPL* subfamilies, along with distinct expression patterns, underscore the complex regulatory landscape of miR156-SPL interactions. These differences not only highlight the functional specialization of SPLs across subfamilies but also suggest that miR156 plays a pivotal role in fine-tuning various agronomic traits through its intricate regulatory network.

The miR156-SPL pathway was initially identified as a key regulator of flowering, primarily involved in the activation of downstream genes associated with the floral transition. However, not all *SPL* genes are linked to age-related pathways, highlighting a nuanced relationship within the *SPL* family. While the expression levels of *SPLs* are regulated by miR156, their own expression also plays a crucial role in this regulatory network. *SPLs* integrate age signals via miR156, emphasizing their significant and conserved function in flowering regulation [7,52,53]. Notably, *CnSPL15A*, identified in coconuts, exhibits a strong correlation with age signals and has been shown to significantly promote the onset of flowering. *CnSPL15A* belongs to subfamily VIII, a key ortholog of *AtSPL9* and *AtSPL15*, known for their roles in regulating the transition from juvenile to adult stages as well as the shift from vegetative to reproductive growth [42,54,55]. Similar functions of *AtSPL9* and/or *AtSPL15* homologs were validated in Lilium [56]. Genomic collinearity between the segments containing CnSPL15A and AtSPL9/AtSPL15 suggests an evolutionary conservation between the two species (Figure S1). In Arabidopsis, overexpression of *CnSPL15A* significantly accelerates flowering and markedly shortens the vegetative growth phase, resulting in a very weak plant phenotype. This suggests that *CnSPL15A* plays a vital role in the age-related regulation of flowering in coconut palms, further illustrating the importance of the miR156-SPL module in plant development. The conservation of this regulatory mechanism across species enhances our understanding of its evolutionary significance and functional diversity.

Coconut, as a perennial woody plant, can continuously flower after entering the reproductive phase. The increased expression of *CnSPL15A* may play a crucial role as a regulatory gene in the transition to reproductive growth in this species. Future research on *CnSPL15A* should focus on several key areas to better understand its functional roles and regulatory mechanisms. One promising avenue is to investigate the specific pathways and molecular interactions mediated by *CnSPL15A* during the transition from vegetative to reproductive growth. This could involve exploring its role in gene expression regulation and identifying downstream targets that contribute to flowering time and plant architecture. Overall, a comprehensive understanding of *CnSPL15A* could not only enhance our knowledge of plant developmental biology but also inform breeding strategies aimed at improving crop yield and resilience in coconut and similar perennial species.

## 5. Conclusions

In conclusion, this study provides significant insights into the roles and characteristics of *CnSPL* genes in coconut palms, highlighting their importance as transcription factors in various biological processes. The classification of 25 *CnSPL* genes into eight subfamilies, along with their conserved structural features and expression patterns, underscores the evolutionary conservation of the *miR156*-*SPL* regulatory module. Notably, while most *SPL* subfamilies include miR156 target sites, variations in gene length and the presence or absence of these sites suggest functional diversification among *SPL* members. Our findings also indicate that *SPL* genes, particularly *CnSPL15A*, are integral to the regulation of flowering age, reinforcing the idea that miR156-mediated regulation is pivotal for plant developmental processes. Furthermore, the presence of unique protein motifs across different subfamilies, alongside the conservation of the miR156 target sequence, illustrates the complex regulatory landscape of *SPL* genes. This research not only enriches the genomic understanding of *CnSPLs* but also lays a foundation for future studies aimed at exploring their roles in agronomic traits and responses to environmental stimuli in coconuts and other species. Overall, the *miR156*-*SPL* pathway emerges as a critical component in the intricate network governing plant growth and development, with potential implications for agricultural practices.

## Data Availability

Data are contained within the article and Supplementary Materials.

## Supplementary Materials

The following supporting information can be downloaded at https://www.mdpi.com/article/10.3390/plants14162532/s1. The information of SPLs genes and primers used in this study were listed in the following tables. Table S1: List of *SPL* genes identified in fifteen plant species. Table S2a: Primers used for RT-qPCR assay of *CnSPLs* in coconut and transgenic Arabidopsis. Table S2b: Primers used for vector construction of *CnSPLs*. Figure S1: Phylogenetic tree and homologous relationships between *CnSPLs* and *AtSPLs*.
