# Correction: Chen et al. Evolutionary Insights and Flowering Regulation of *SPLs* in Coconut Palm. *Plants* 2025, *14*, 2532

**DOI:** 10.3390/plants14243752

**Published:** 2025-12-10

**Authors:** Runan Chen, Yalan Feng, Jin Zhou, Ying Wang, Fengyi Zhang, Shazia Rehman, Zhuang Yang, Zifen Lao, Hang Xu, Yong Xiao, Jie Luo, Wei Xia

**Affiliations:** State Key Laboratory of Topical Crop Breeding, School of Breeding and Multiplication (Sanya Institute of Breeding and Multiplication)/School of Tropical Agriculture and Forestry, Hainan University, Sanya 572025, Chinajie.luo@hainanu.edu.cn (J.L.)

**Error in Figure**

In the original publication [1], there was a mistake in Figure 2 as published. The annotation for the miR156-target sequence sites on CnSPL is missing from Figure 2. The corrected Figure 2 appears below. The authors state that the scientific conclusions are unaffected. This correction was approved by the Academic Editor. The original publication has also been updated.


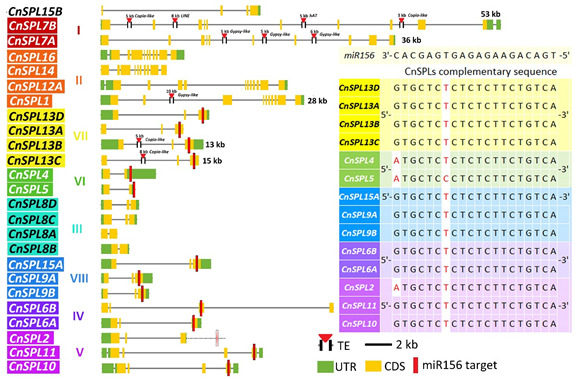


## References

[1] Chen R., Feng Y., Zhou J., Wang Y., Zhang F., Rehman S., Yang Z., Lao Z., Xu H., Xiao Y. (2025). Evolutionary Insights and Flowering Regulation of *SPLs* in Coconut Palm. Plants.

