# Supplementary figures and images for: Evolutionary Insights and Flowering Regulation of SPLs in Coconut Palm
Source: Plants (Basel). 2025 Aug 14;14(16):2532. doi: 10.3390/plants14162532 (PMC12389045; doi:10.3390/plants14162532)

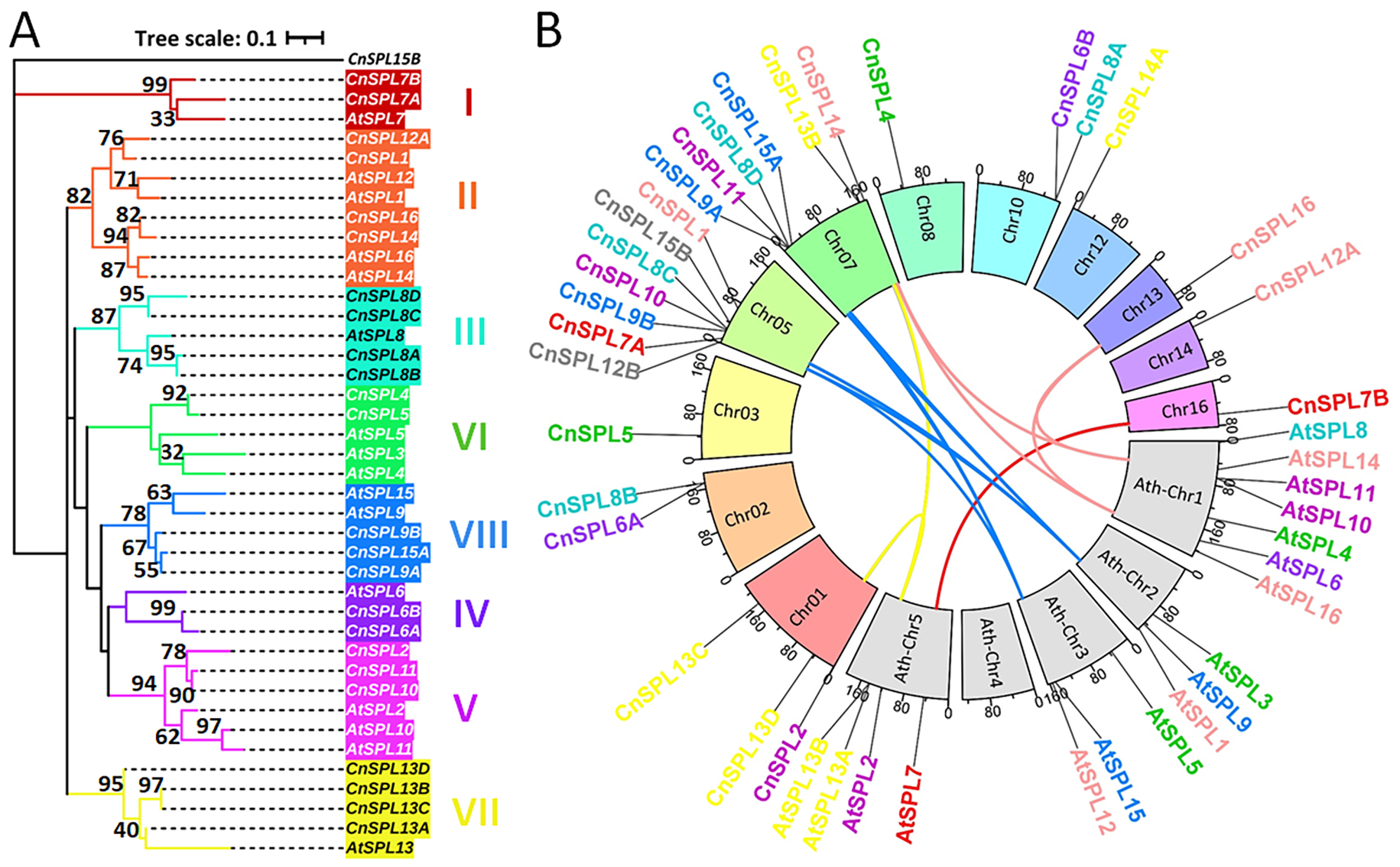

Supplement: Supplementary file 1 [file plants-14-02532-s001.zip › Supplementary file/Figure S1.jpg]
